# A New Compound Staged Gelling Acid Fracturing Method for Ultra-Deep Horizontal Wells

**DOI:** 10.3390/gels8070449

**Published:** 2022-07-18

**Authors:** Yang Wang, Yu Fan, Tianyu Wang, Jiexiao Ye, Zhifeng Luo

**Affiliations:** 1 Engineering Technology Research Institute of Southwest Oil & Gas Field Company, PetroChina, Chengdu 610017, China; fanyu@petrochina.com.cn (Y.F.); yejiexiao@petrochina.com.cn (J.Y.); 2 Southwest Oil & Gas Field Company, Petrochina, Chengdu 610017, China; wty01@petrochina.com.cn; 3 State Key Laboratory of Oil and Gas Reservoir Geology and Exploitation, Southwest Petroleum University, Chengdu 610500, China; lzfswpu2011@163.com

**Keywords:** compound, staged, gelling acid fracturing, temporary plugging

## Abstract

Carbonate gas reservoirs in Sichuan are deeply buried, high temperature and strong heterogeneity. Staged acid fracturing is an effective means to improve production. Staged acidizing fracturing of ultra-deep horizontal wells faces the following problems: 1. Strong reservoir heterogeneity leads to the difficulty of fine segmentation; 2. The horizontal well section is long and running too many packers increases the completion risk; 3. Under high temperatures, the reaction speed between acid and rock is rapid and the acid action distance is short; and 4. The fracture conductivity is low under high-closure stress. In view of the above problems, the optimal fracture spacing is determined through productivity simulation. The composite temporary plugging of fibers and particles can increase the plugging layer pressure to 17.9 MPa, which can meet the requirements of the staged acid fracturing of horizontal wells. Through the gelling acid finger characteristic simulation and conductivity test, it is clear that the crosslinked authigenic acid and gelling acid in the Sichuan carbonate gas reservoir are injected alternately in three stages. When the proportion of gelling acid injected into a single section is 75% and the acid strength is 1.6 m^3^/m, the length and conductivity of acid corrosion fracture are the best. A total of 12 staged acid fracturing horizontal wells have been completed in the Sichuan carbonate gas reservoir, and the production is 2.1 times that of ordinary acid fracturing horizontal wells.

## 1. Introduction

Sichuan carbonate gas reservoir is rich in natural gas resources. The reservoir has the characteristics of high temperature, low porosity, and low permeability [1,2,3]. The formation temperature can reach 160 °C, and the reservoir lithology is mainly dolomite. The gas production of vertical wells after fracturing is low, which cannot support the economic development of gas reservoirs [4,5]. Staged acid fracturing of horizontal wells is a favorable means to improve the development effect of gas reservoirs [6,7,8]. Staged fracturing technologies mainly comprise double-pack single-slip multistage fracturing [9], packer-sliding sleeve fracturing [10], and hydraulic jetting multi-stage fracturing [11]. Staged acid fracturing of ultra-deep horizontal wells faces many problems, such as fracture parameter optimization, staged fracturing process optimization, and acid corrosion fracture length improvement.

Productivity simulation and fracture parameter optimization of horizontal wells are the basis of staged acid fracturing of horizontal wells [12,13,14]. Simple analytical solutions are derived on the basis of the assumption of infinite drain hole conductivity. These models are widely adopted because they are easy to use. However, they are not able to produce an accurate prediction of productivity because they ignore the pressure drop in the wellbore [15,16,17,18]. Hemanta Mukherjee et al. [19] conducted a series of studies about the productivity of fractured horizontal wells in a uniform sandstone reservoir. Yang He et al. [20] established the productivity-prediction model, which was suitable for the acid fracturing horizontal well in the fracture-cavity reservoir, by comprehensively considering primary factors, wellbore pressure drop, cracks for supporting liquid, and connected fracture system.

The reaction rate of acid liquid rises sharply at high temperatures, which seriously affects the acid fracturing effect of ultra-deep reservoirs [21,22,23]. Settari [24] developed a comprehensive acid fracturing model that solved for 2D/pseudo-3D fracture geometry, leakoff, heat transfer, and acid transport. Rencheng Dong et al. [25] developed a 3D acid fracturing model to compute the rough acid fracture geometry induced by multi-stage alternating injection of pad and acid fluids, they investigated the effects of viscous fingering, perforation design, and stage period on the acid etching process. S.J. Jackson et al. [26] investigated the stability of immiscible viscous fingering in Hele-Shaw cells with spatially varying permeability, providing an efficient, high accuracy scheme to track the interfacial displacement of immiscible fluids. Tu Nguyen et al. [27] developed an improved VOF interface-sharpening method on the general curvilinear grid for two-phase flows. Haoran Xu et al. [28] developed a modeling framework for the coupled thermal-hydro-mechanical chemical processes during acid fracturing in carbonatite geothermal reservoirs. Jiahui You et al. [29] developed a pore–scale numerical model to analyze the impact of acid–rock interaction on multiphase flow behavior, and the pore–scale model in this research provides the fundamental knowledge of the physical and chemical phenomena of acid–rock interactions and their impact on acid transport. Hossein Mehrjoo et al. [30] studied the final fracture conductivity during acid fracturing. Xu P et al. [31] studied the influences of rock microstructure on acid dissolution on a dolomite surface. Daobing Wang et al. [32] used a cohesive zone method (CZM)-based finite element model to obtain the fracture closure pressure and minimum horizontal principal stress of the major fracture and the branch fracture based on pressure fall-off analysis.

Deep high-temperature vuggy naturally fractured formations where tools are not reliable and costive [33], and diverted fracturing with temporary plugging has become an option [34]. Degradable fiber is another commonly used diverter. It can bridge on the fracture, or fill the gap between particulate diverters to form a tight barrier. A combination of particulate diverter and fiber was often applied in the fields [35,36]. Jianye Mouet et al. [37] designed a multi-stage triaxial fracturing system and experimental procedures to satisfy the requirements of diverted fracturing in horizontal wells. Among the various existing chemical diverters, temporary plugging agents have the greatest potential for being used in HPHT reservoirs. Temporary plugging agents are made from degradable polymers, typically polylactic acid (PLA), which can degrade at the reservoir temperature and leave no residue [38]. All reported field applications with this type of diverter are in reservoirs less than 100 °C, and only used for plugging the perforation holes during the refracturing [39,40,41]. Zhou et al. [42] investigated the filtration characteristic of temporary plugging and the length of filter cake under the condition of different fracture widths. Daobing Wang et al. [43] presented a comprehensive workflow to model hydraulic fracture by accounting for interactions with numerous crosscutting natural fracture or joint sets, as well as the effect of temporary plugging in opened fractures. This model is a fully coupled seepage flow in porous media, fluid flow in fractures, and rock deformation finite element model with adaptive insertion of cohesive elements as a crosscutting natural fracture or joint sets. Wang Mingxing et al. [44] designed a high-pressure evaluation system for fracture temporary plugging and investigated the key factors and their influencing pattern. Chen Yang et al. [45] conducted a series of experiments to investigate the plug formation time, diverter consumption, and plugging zone characteristics with different fracture widths and diverter concentrations. Zhu Baiyu et al. [46] established the visualization model of a microfluidic chip and CFD–DEM numerical simulation to evaluate the effect of particle plugging in a fracture-vuggy reservoir.

According to the previous studies, it can be concluded that there are still many technical problems in the staged acid fracturing of ultra-deep horizontal wells. Therefore, this paper introduces a new compound staged gelling acid fracturing method for ultra-deep horizontal wells. Using productivity simulation technology, we optimize the optimal fracture spacing and fracture length of segmented acid fracturing in horizontal wells. Through dissolution and plugging pressure tests of the temporary plugging agent, the combination of high-strength temporary plugging agent is optimized, and the key parameters of alternating injection acid fracturing process are optimized by the VOF model. This study deepens the understanding of the staged acid fracturing mechanism in an ultra-deep horizontal well, and provides great help for acid fracturing stimulation design. The compound staged gelling acid fracturing technology is widely used in ultra-deep horizontal wells in the Sichuan Basin, which has achieved a good stimulation effect and improved the development effect of gas reservoirs.

## 2. Results and Discussion

**Figure 1:**
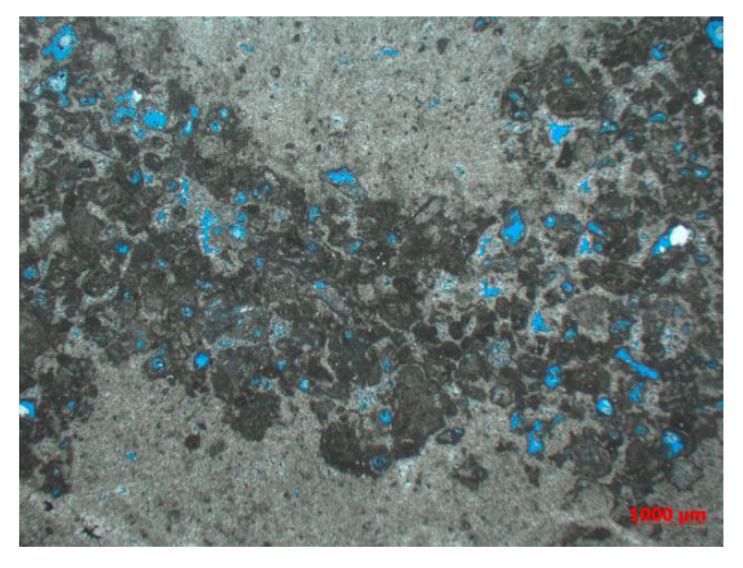
Thin section of well QY1.

The buried depth of the carbonate gas reservoir in the Sichuan Basin is 6 to 7 km and the temperature is 140 to 160 °C. It is mainly composed of biological micrite ash and algal agglomerate dolomite. There are various types of gas reservoir space, including intergranular pores, intragranular pores, and intergranular pores (Figure 1), as well as karst caves, solution fractures, and structural fractures. The average porosity of the reservoir is 3.4% and the average permeability is 0.4 mD. In terms of natural fractures, they are mainly structural fractures. The section of structural fractures is generally straight, mostly with high-angle fractures. The wall of dissolution fractures is uneven and harbor shaped, and the structural dissolution fractures are generally filled with asphalt or dolomite. The core observation shows that the fractures are relatively developed, the fracture density is 2.86 pieces/m, and the connectivity between the fractures and various effective pores is good. According to the comparison of reservoir vertical development characteristics of multiple wells, the thickness of a single well reservoir is thin, and the average thickness is only 11.8 m.

**Figure 2:**
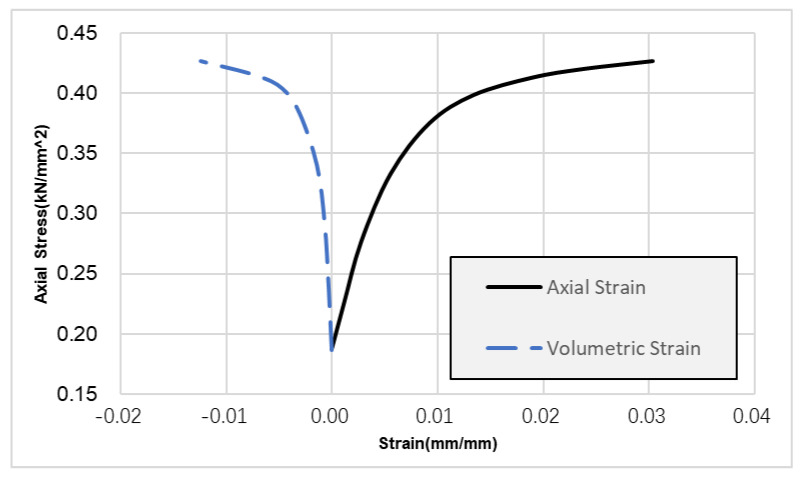
Stress−strain curve of well QY32.

According to the core test, the average Young’s modulus of the carbonate reservoir in the Sichuan Basin is as high as 60 GPa and the average compressive strength is 510 Mpa. The high Young’s modulus and compressive strength lead to the narrow width of acid fracturing fracture. The Poisson’s ratio of the reservoir is 0.29. We used a GCTS RTR-2000 tester to evaluate the rock mechanical properties of cores in the Sichuan Basin, and it can be observed from Figure 2 that, with the increase in axial stress, there is no inflection point in the axial strain and volumetric strain curves, indicating that the reservoir cores in the Sichuan Basin have typical plastic characteristics, so it is difficult to maintain the conductivity of acid corrosion fractures under high closure pressure. Carbonate reservoirs in the Sichuan Basin have obvious plastic characteristics. Because the plastic deformation of rocks needs to consume additional energy, the fracture extension needs high pressure. Under the condition of the same injection displacement, the fracturing fracture formed in the elastic–plastic formation is relatively short and wide.

The differential strain test results show that the maximum horizontal principal stress of the reservoir is 170.2 MPa, the minimum horizontal principal stress is 148.1 MPa, the vertical principal stress is 200.8 MPa, and the horizontal two-way stress difference is 22.1 MPa, and the horizontal stress difference coefficient is 0.15. According to the distribution law of three-way principal stress, the fractures formed by acid fracturing of carbonate rock reservoir in the Sichuan Basin are mainly vertical fractures.

**Figure 3:**
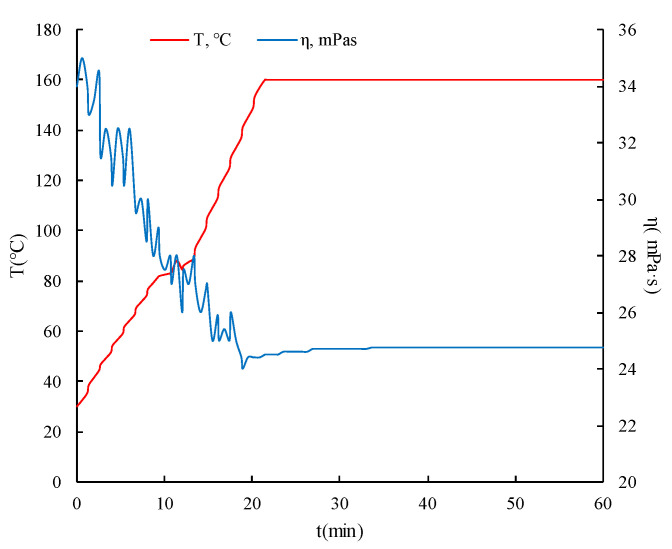
Stress–strain curve of well QY32.

Gelling acid is the most commonly used acid system for acid fracturing of gelling acid carbonate gas reservoir. Gelling acid has the characteristics of low friction and low acid-rock reaction speed. The gelling agent of acid is a linear high-molecular polymer. After being fully dissolved in acid solution, the molecular chains stretch and entangle with each other to form a spatial network structure and produce structural viscosity. The mechanical and thermal properties of gelled acid were evaluated by HAAKE rheometer, its density was 1.1 g/cm^3^ and its viscosity was 24 mPa·s after shearing for 60 min at 160 °C (Figure 3). Gelling acid can maintain good viscosity at high temperatures, which is conducive to acid fracturing stimulation.

### 2.1. Optimal Fracture Parameters of Acid Fracturing for Horizontal Wells

The optimization of fracture spacing is the key to the optimization of acid fracturing design [47,48,49]. Reasonable fracture spacing can reduce unnecessary packers and mutual interference between acid fracturing fractures. Reducing the fracture spacing is conducive to expanding the drainage area of gas wells, but the research shows that, when the fracture spacing is shortened to a certain extent, the productivity of gas wells will not be significantly improved, and there is an economic optimal fracture spacing for staged acid fracturing of horizontal wells [50,51,52,53]. According to the reservoir porosity, permeability, gas saturation, and other physical parameters in the Sichuan Basin, we used Eclipse to establish the numerical simulation model of staged acid fracturing horizontal wells in carbonate gas reservoirs, and the relevant parameters selected in the model are shown in Table 1.

**Figure 4:**
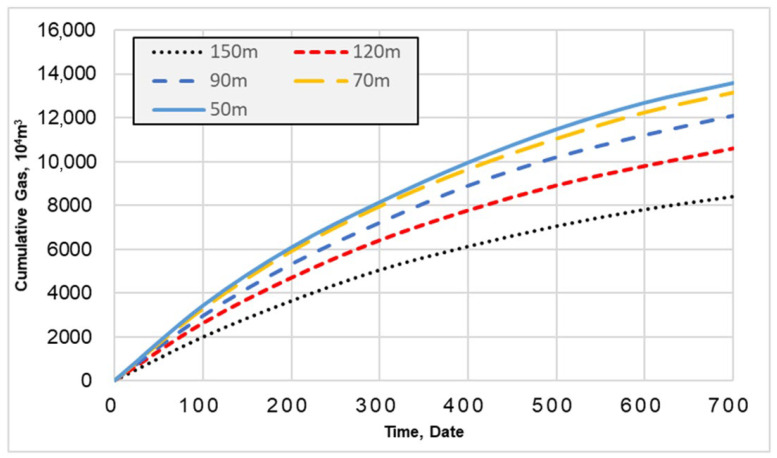
Productivity simulation under different fracture spacing.

It can be observed from Figure 4 that the gas production of horizontal wells increases significantly with the decrease in fracture spacing. However, when the fracture spacing is reduced to 50 m, the gas production of horizontal wells is only 5.3% higher than that when the fracture spacing is 70 m, and the gas production does not significantly increase. Therefore, the optimal fracture spacing for the segmented acid fracturing of horizontal wells in carbonate gas reservoirs in the Sichuan Basin is 70 m.

### 2.2. Packer + Temporary Plugging

Staged acid fracturing technology of packer is an important measure to increase the production of the carbonate reservoir [54]. Its process is to run multiple packers in an open-hole horizontal well at one time, and divide the horizontal well section into several independent well sections for acid fracturing through the packer. The length of the horizontal section of the horizontal wells in carbonate gas reservoirs in the Sichuan Basin can reach 1.2 km, and the optimal fracture spacing according to productivity simulation is 70 m. If we only use packers for staged acid fracturing, we need to run at least 18 packers in the well to fully stimulate the formation, but running too many packers not only increases the risk of well completion [55], it also leads to greatly increases the throttling pressure, thus affecting the injection rate [56]. Through calculations, the carbonate gas reservoir in the Sichuan Basin only adopts the packer, which increases the throttling pressure by at least 30 MPa, and the acid injection rate is only 2 m^3^/min. From such a low injection rate it is difficult to obtain long acid corrosion fractures at high temperature. In this paper, a new compound staged acid fracturing method was proposed to solve the problem of difficult stimulation of long interval horizontal wells. The goal of this method is to achieve tight cutting stimulation while reducing packers.

**Figure 5:**
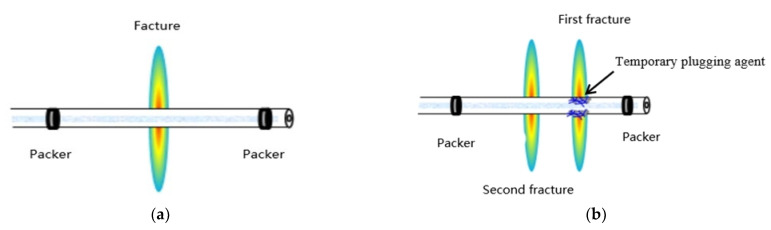
Schematic diagram of the staged acid fracturing technology. (**a**) Packer staged acid fracturing; (**b**) “Packer + temporary plugging” staged acid fracturing.

Aiming at the problem of difficult stimulation in the long well section, a new compound staged acid fracturing method is proposed in this paper. The goal of this method is to stimulate the reservoir while reducing the packer. The process principle is to use the temporary plugging agent to replace some packer functions. After throwing the ball to open the sliding sleeve, we first injected liquid to create the first fracture in the formation, then added the temporary plugging agent to block the end of the first fracture, and finally injected liquid for the second fracturing. This stimulation method can not only ensure the optimal fracture spacing, but also reduce the packer by half (Figure 5).

The reservoir temperature in the Sichuan Basin is high. The first requirement of acid fracturing for the temporary plugging agent is that the dissolution rate of the temporary plugging agent in a high-temperature acid liquid cannot be too high, because if the temporary plugging agent dissolves quickly, there is no way to form a stable plugging layer in the formation. This paper evaluated the dissolution experiments of temporary plugging agents of different materials at 150 °C, and the experiment shows that the dissolution rate of the polyemulsion-modified polyvinyl alcohol resin is low at 150 °C (Table 2), but the dissolution rate of the temporary plugging agent of other materials is more than 50%. The polyemulsion-modified polyvinyl alcohol resin with a low dissolution rate was selected for the temporary plugging acid fracturing in the Sichuan Basin. The polyemulsion-modified polyvinyl alcohol resin used in the experiment came from the Chengdu LEPS technology company; its molecular weight was 180,000–240,000 and it was completely dissolved in water after 3 h at 150 °C. Urea methyl ester is the urea formaldehyde resin. It is the condensation polymerization of urea and formaldehyde under the action of a catalyst to form the initial urea formaldehyde resin, and then form the thermosetting resin under the action of the curing agent. The molecular formula of polyethylene glycol is HO(CH_2_CH_2_O)_n_H.

The reservoir in the Sichuan Basin is highly heterogeneous. According to statistics, the maximum fracture pressure difference between points in the horizontal section is 15 MPa. Therefore, in order to achieve an effective temporary plugging acid fracturing, the plugging layer formed by the temporary plugging agent must bear a pressure of at least 15 MPa. Fibers and particles are commonly used temporary plugging materials [57]. The plugging principle is to achieve a high-strength plugging through particle bridging and fiber winding. The polyemulsion-modified polyvinyl alcohol resin was processed into fibers with a length of 6–8 mm and particles with different particle sizes. In this paper, the plugging ability of fibers and particles was evaluated using a three-dimensional fracture dynamic plugging experimental equipment. By placing fibers and particles into clean water in a certain proportion and driving them into the simulated acid fracturing fracture at a uniform speed with a precision pressure pump, the displacement pressure change of the temporary plugging material after entering the simulated fracture was observed with an electronic pressure gauge.

To carry out the fracture plugging test with the temporary plugging agent, the first step was to determine the fracture width of the temporary plugging agent. Through the simulation of fracturing software, it was found that the average width of acid corrosion fracture in the Sichuan carbonate gas reservoir is 6 mm. Therefore, the fracture width of this test was determined as 2 mm and the test temperature was 150 °C.

**Figure 6:**
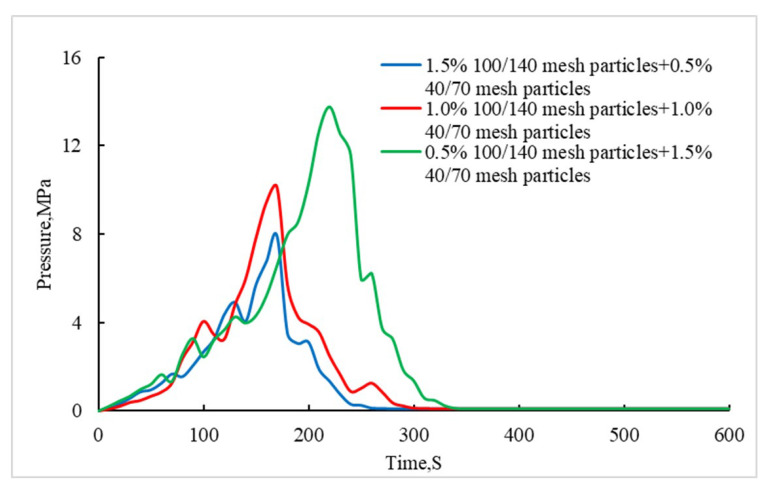
Comparison of the fracture sealing ability of the combination of differently sized particles.

It can be seen from Figure 6 that, after adding 40/70 mesh particles to 100/140 mesh particles, the pressure bearing capacity of the plugging layer is significantly improved. Especially when the concentration of 40/70 mesh particles increases to 1.5%, the pressure bearing capacity of the plugging layer can be increased to 13.7 MPa. Therefore, the addition of large particles is helpful to quickly bridge and form a sealing plugging layer. The pressure of the plugging layer is required to be higher than 15 MPa for the temporary plugging acid pressure of the Sichuan carbonate gas reservoir. Therefore, we considered adding an appropriate concentration of fibers into the particles and filling the gap between the particles of different sizes with fiber, so as to further improve the pressure bearing capacity of the plugging layer.

**Figure 7:**
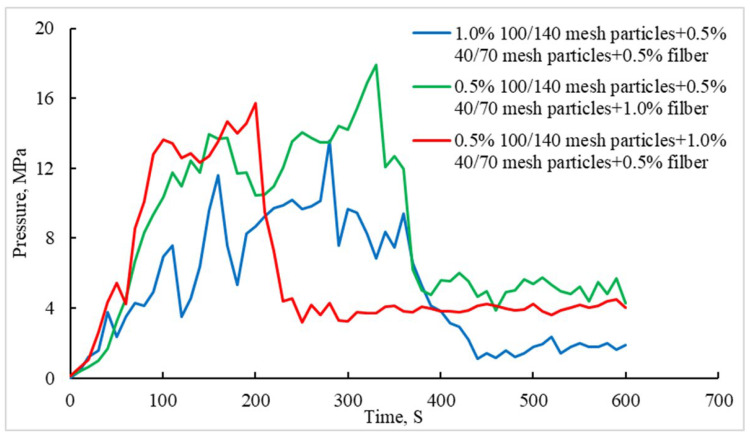
Comparison of the fracture sealing ability of the particle and fiber combination.

**Figure 8:**
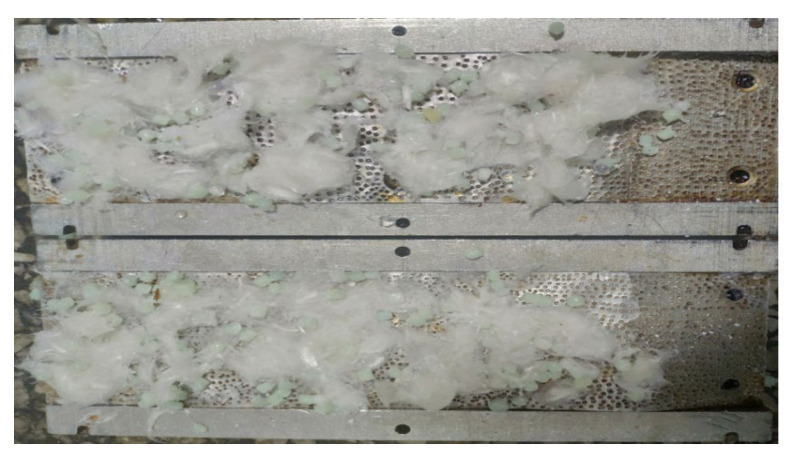
Distribution of the temporary plugging materials in a simulated fracture.

It can be seen from Figure 7 that, after adding fiber to particles, the pressure bearing capacity of the plugging layer is significantly improved. Especially when the fiber concentration reaches 1.0%, the pressure bearing capacity of the plugging layer can be increased to 17.9 MPa, which is 4.2 MPa higher than that of the plugging layer with only particles. The fiber + particle composite temporary plugging method meets the requirements of the staged acid fracturing of the Sichuan carbonate gas reservoir. It can be seen from Figure 8 that fibers and particles almost fill the whole simulated fracture, 40/70 mesh particles are distributed in a scattered manner, fibers and 100/140 mesh particles are intertwined to form a dense layer, and they fill the gap between the 40/70 mesh particles.

The sealing layer is composed of fibers and particles. The particles quickly accumulate in the fracture to form a sealing layer. At the same time, the fibers are intertwined to block the gap between the particles, so as to improve the pressure bearing capacity of the sealing layer. As can be seen from Figure 7, with the increase in the fiber dosage, the peak pressure bearing capacity of the plugging layer increases significantly.

### 2.3. Acid Fracturing Technology for the Ultra-Deep Reservoir

A high-temperature carbonate reservoir has a fast acid rock reaction speed and short acid corrosion fracture length [58]. Alternating acid fracturing injection is an effective means to improve the length of the acid fracturing fracture. The fingering phenomenon is produced by injecting liquids with different viscosities, which not only improves the effective action distance of acid, but also forms non-uniform etching on the fracture wall to further improve the conductivity [59]. Based on the multiphase flow VOF model and the test results of acid corrosion fracture conductivity, the acid fracturing process of alternating injection in the Sichuan carbonate gas reservoir was studied, and the key parameters, such as the optimal injection liquid viscosity ratio, injection displacement, and scale, were determined.

**Figure 9:**
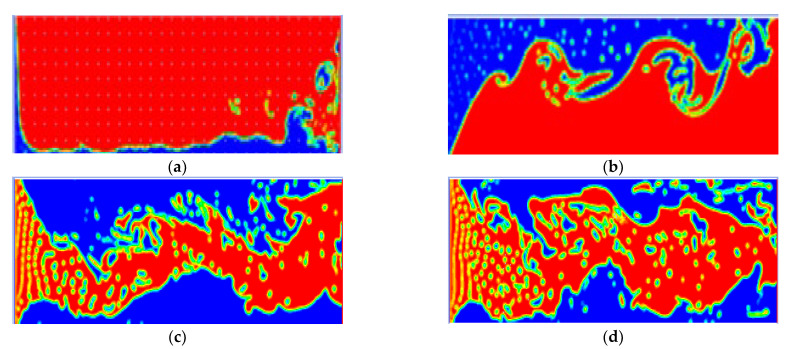
Fingering form of the acid solution under different viscosity ratios (red represents gelling acid and blue represents crosslinked authigenic acid). (**a**) Viscosity ratio 1:1; (**b**) Viscosity ratio 3:1; (**c**) Viscosity ratio 8:1; and (**d**) Viscosity ratio 12:1.

It can be seen from Figure 9 that, with the increase in the viscosity ratio of the two liquids, the fingering characteristics of acid liquid in the fracture become more and more obvious, and the flow pattern of acid liquid evolves from uniform piston propulsion to narrow fingering propulsion. When the viscosity ratio of fracturing fluid (blue part) to acid (red part) increases to 8:1, the fingering pattern of acid liquid tends to be stable. The non-uniform distribution of acid solution is conducive to strengthening the differential acid corrosion of rock and plays a positive role in maintaining and improving the fracture conductivity in the later production stage [59]. The viscosity of crosslinked autogenous acid is 150 mPa·s and the viscosity of gelling acid is 18 mPa·s. The viscosity ratio of the above two liquids is slightly greater than 8:1. The crosslinked authigenic acid and gelling acid meet the requirements of the best viscosity ratio of the alternating injection.

**Figure 10:**
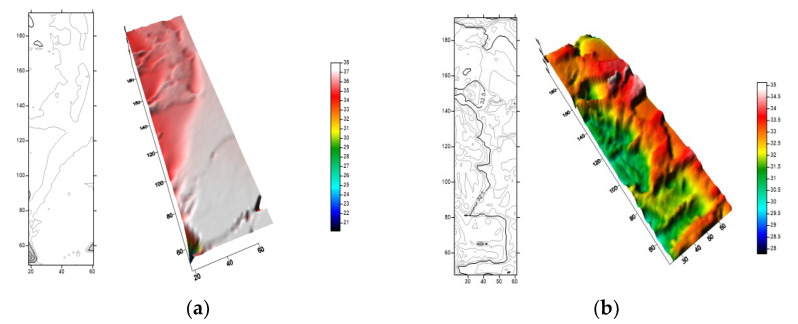
Acid corrosion fracture wall after injecting acid with different viscosities. (**a**) Viscosity ratio 3:1; and (**b**) Viscosity ratio 8:1.

We selected two carbonate rock plates with the same mineral composition for the experiments and compared the surface morphology of the fractures by injecting acids with different viscosities. It can be seen from Figure 10 that the differential corrosion ability of acid is stronger after increasing the viscosity ratio of the acid solution. The fracture corrosion effect observed in the acid etching experiment is the same as that of the numerical simulation, which further proves the accuracy of the numerical simulation in the simulation of the acid fingering behavior.

**Figure 11:**
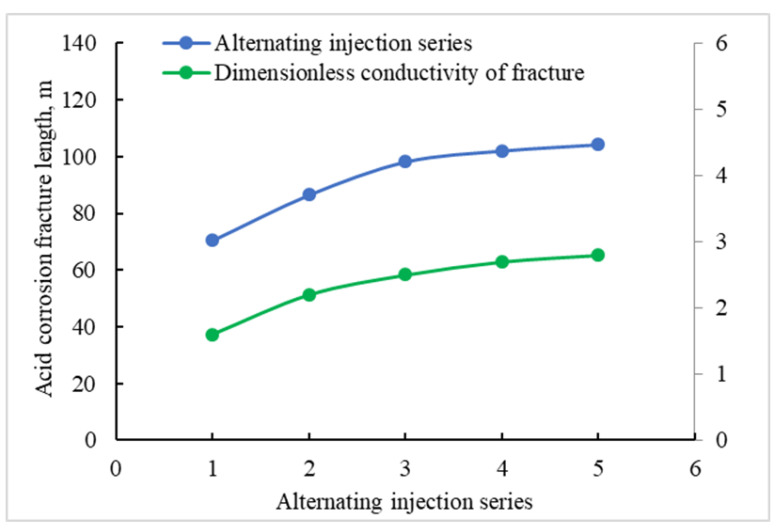
Acid corrosion fracture parameters under different injection series.

We Kept the displacement and scale of the acid injection unchanged, and simulated the length of the acid corrosion fracture and dimensionless conductivity under the condition of series 1 to 5 injection. As can be seen from Figure 11, with the increase in the injection series, the length of acid corrosion fracture and dimensionless conductivity rise synchronously. When the alternating injection series is 3, the growth range of acid corrosion fracture length and dimensionless conductivity slows down. Compared with a three-stage alternate injection, the length of acid corrosion fracture of the five-stage alternate injection increases by only 6 m, with an increased rate of 6.12%. The best series of alternating injection acid fracturing in the Sichuan carbonate gas reservoir is 3, and the length of acid corrosion fracture can reach 99.6 m.

**Figure 12:**
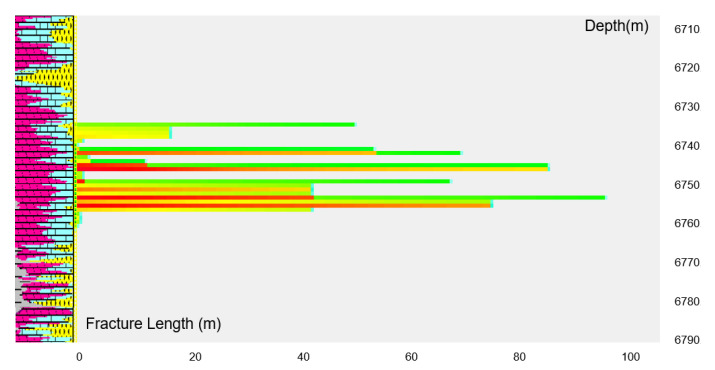
Simulation diagram of the acid corrosion fracture width under the three-stage alternating injection. (Red represents fractures with a width of 7–9 mm, yellow represents fractures with a width of 5–7 mm, and green represents fractures with a width of 3–5 mm).

**Figure 13:**
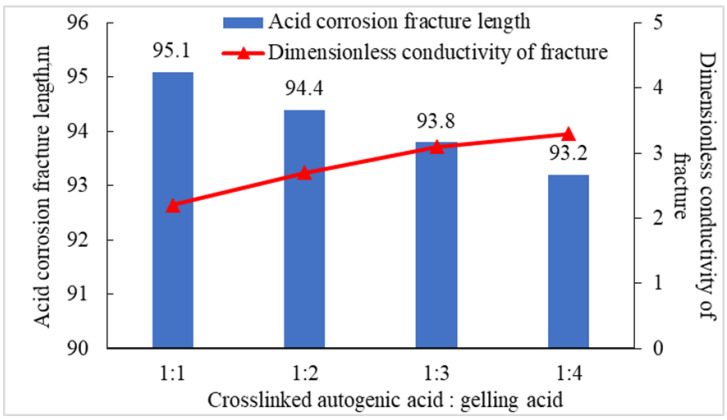
Acid corrosion fracture parameters under different liquid injection ratios.

We used the acid fracturing software to simulate the fracture shape of alternating injection acid fracturing. The physical model was established according to the actual geological and mechanical parameters of carbonate rocks in the Sichuan Basin. The acid corrosion fracture shape was simulated according to the three-stage alternating injection of authigenic acid and gelling acid with an injection displacement of 7 m^3^/min. Figure 12 is the simulation diagram of the acid corrosion fracture width. The color represents the width of the acid corrosion fracture, and the dark color shows that the width of the acid corrosion fracture is large. It can be seen from Figure 13 that the width of the acid corrosion fracture changes greatly after a three-stage alternating injection of acid fracturing. The multiple alternating injection of the crosslinked authigenic acid and gelling acid intensifies the interface effect between liquids and promotes the generation of the acid fingering phenomenon.

Under the condition of keeping the injection displacement, injection stages, and total amount of liquid unchanged, the fracturing software was used to simulate the acid fracturing fracture extension under different liquid injection ratios. It can be seen from Figure 13 that, with the decrease in the amount of crosslinked autogenous acid, the length of acid fracturing fracture decreases, and the conductivity of acid etching fracture increases steadily. When the volume ratio of the crosslinked authigenic acid and gelling acid is 1:3, the length of acid etching fracture is close to 100 m, and the dimensionless conductivity of fracture is close to the maximum. The acid fracturing design of the Sichuan carbonate gas reservoir takes into account the two parameters of fracture length and conductivity, and the volume ratio of the injected crosslinked authigenic acid and gelling acid is preferably 1:3.

**Figure 14:**
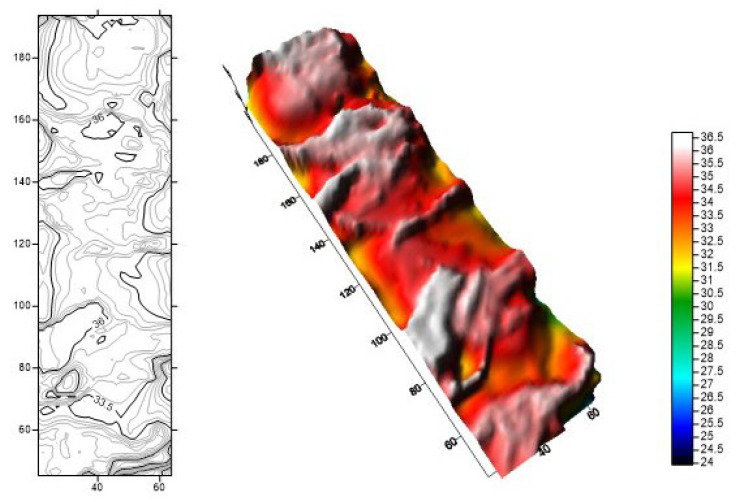
Wall morphology of the acid corrosion fracture.

As can be seen from Figure 14, when the injection volume ratio of the crosslinked autogenous acid and gelling acid is 1:3, the acid liquid creates non-uniform corrosion on the rock wall, the acid liquid creates a groove corrosion with a certain height difference on the rock wall, and the non-corrosion rock creates a support resembling a pier, which improves the effectiveness of the crack under high closure stress.

**Figure 15:**
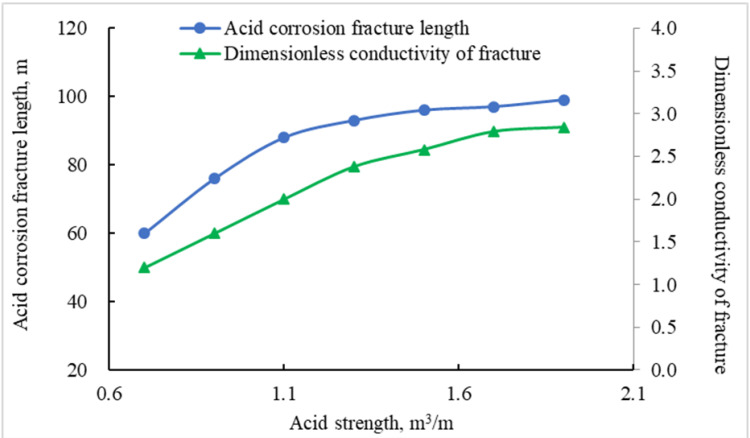
Simulation results of the acid corrosion fracture parameters under different acid strengths.

Acid strength refers to the amount of acid used per meter of the reservoir during acid fracturing. The acid corrosion fracture length and dimensionless conductivity when the acid strength is from 0.7 to 1.9 m^3^/m were simulated by fracturing software. It can be seen from Figure 15 that, with the increase in acid strength, the acid corrosion fracture length and dimensionless conductivity increase rapidly because increasing the amount of acid can ensure that more acid liquid reacts with the rock, so as to improve the acid fracturing effect. When the acid strength increases to 1.6 m^3^/m, the growth rate of the acid corrosion fracture length and dimensionless conductivity slows down. When the acid strength is increased to 2.0 m^3^/m, the length of acid corrosion fracture is 2.8 m longer than that of 1.6 m^3^/m. The optimal acid strength of the Sichuan carbonate gas reservoir is 1.6 m^3^/m, which can not only ensure a better fracture length and conductivity, but also reduce the excessive consumption of acid liquid and improve the economy of the acid fracturing stimulation.

### 2.4. Field Application

In the Sichuan carbonate gas reservoir, a total of 12 horizontal wells have been stimulated by staged acid fracturing, and the production is 2.1 times higher than that of general stimulation. Among them, the drilling depth of well QY1019 is 6823 m, the horizontal section length is 1185 m, the reservoir section length is 901 m, the average porosity of the reservoir is 3.2%, and the average permeability is 0.31 mD. In 2021, the QY1019 well completed the compound staged acid fracturing stimulation through packer + temporary plugging. A total of 480 m^3^ authigenic acid, 1440 m^3^ gelling acid, 800 kg fiber, and 800 kg temporary plugging particles were injected into the formation. In order to verify the effectiveness of staged acid fracturing for the temporary plugging, two kinds of chemical tracers were added before and after the injection of the temporary plugging agent. The chemical tracer interpretation results showed that new fractures were formed after the addition of a temporary plugging agent. Net pressure fitting shows that, compared with conventional acid fracturing, the fracture length and conductivity of alternating injection acid fracturing increased by 78.3% and 52.5% respectively. The daily gas production is 1.58 × 10^4^ m^3^/d, which is 2.4 times of the test daily gas production of the adjacent wells.

## 3. Conclusions

In this study, we proposed a new staged acid fracturing method for ultra-deep horizontal wells, which uses the packer and the temporary plugging agent to realize the uniform stimulation of long well sections. We used numerical simulation software to determine that the optimal fracture spacing of horizontal wells in the carbonate gas reservoirs in the Sichuan Basin is 70 m. Through the pressure test of the plugging layer, the composite temporary plugging mode of fiber and particle was proposed. The fiber and particle can form a plugging layer with a pressure bearing capacity of more than 17 MPa, which meets the stimulation requirements. Through the simulation of gelling acid fingering form and the test of conductivity, it was found that the best acid fracturing effect can be obtained by injecting crosslinked autogenous acid and gelling acid alternately in three stages and increasing the amount of acid. This study has great guiding significance for the stimulation of ultra-deep carbonate gas reservoirs.

## Figures and Tables

**Table 1:** Numerical simulation-model parameters.

Grid side length (m)	0.1	Reservoir permeability (mD)	0.308
Model I-axis length (m)	3000	Gas saturation (%)	86.2
Model J-axis length (m)	600	Fracture conductivity (D·cm)	14
Model K-axis length (m)	12	Rock compressibility	3.9 × 10^−7^
Reservoir porosity (%)	3.12	Formation pressure (MPa)	98.2

**Table 2:** Experiment on the solubility of the temporary plugging agents with different materials.

Experimental Sample	Dissolution Rate of Test Sample in 20% Hydrochloric Acid at 150 °C
Polyemulsion-modified polyvinyl alcohol resin	3.54%
Urea methyl ester	92.04%
Modified polyethylene glycol	96.65%

## Data Availability

Not applicable.
